# Supplementary material for: Spatial Modelling to Inform Public Health Based on Health Surveys: Impact of Unsampled Areas at Lower Geographical Scale
Source: Int J Environ Res Public Health. 2020 Jan 28;17(3):786. doi: 10.3390/ijerph17030786 (PMC7036870; doi:10.3390/ijerph17030786)
Supplement: Supplementary file 1 [file ijerph-17-00786-s001.zip › Final_IJERPH_Supplementary_Files.pdf]

# Supplementary Materials

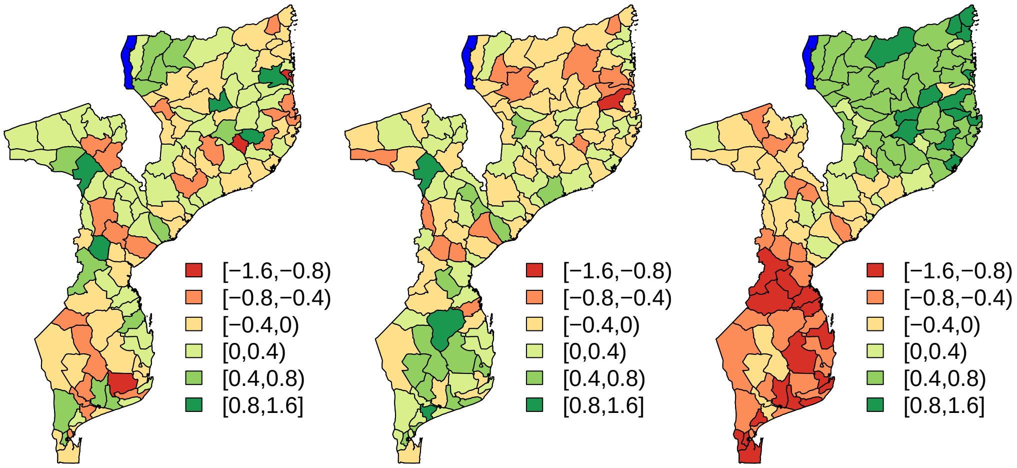


**Figure 1.** Simulation 1: Plot of generated random effects $u_k + v_k$ for a weak spatial trend (left), moderate spatial trend (middle) and strong spatial trend (right).

| (B1) | | (B2) | |
|---|---|---|---|
| Parameter | Estimate | Parameter | Estimate |
| $\hat{\alpha}$ | 0.79 | $\hat{\alpha}$ | 0.71 |
| | | $\hat{\beta}_{candle}$ | 0.40 |
| | | $\hat{\beta}_{electricity}$ | 1.39 |
| | | $\hat{\beta}_{firewood}$ | -0.33 |
| | | $\hat{\beta}_{gas}$ | 1.42 |
| | | $\hat{\beta}_{generator/solarpower}$ | 1.01 |
| | | $\hat{\beta}_{oil/kerosene/paraffin}$ | -0.07 |
| | | $\hat{\beta}_{other}$ | -0.41 |

**Table 1.** Simulation 2: Parameter values for the true $\alpha$- and $\beta$- parameters in scenario's (B1) and (B2)
.

| # Missing Districts | UNW | HT | NB | LN | AS | ES |
|---|---|---|---|---|---|---|
| Bias$^2$ ($\times 10^3$) | | | | | | |
| 0 | 0.03 | 0.03 | 0.71 | 0.86 | 0.63 | 0.74 |
| 5 | 0.03 | 0.03 | 0.71 | 0.85 | 0.63 | 0.74 |
| 10 | 0.03 | 0.03 | 0.76 | 0.91 | 0.68 | 0.8 |
| 15 | 0.03 | 0.03 | 0.87 | 1.03 | 0.78 | 0.91 |
| 20 | 0.04 | 0.04 | 0.89 | 1.07 | 0.8 | 0.93 |
| 25 | 0.03 | 0.03 | 0.91 | 1.11 | 0.81 | 0.95 |
| 30 | 0.05 | 0.05 | 0.87 | 1.04 | 0.76 | 0.91 |
| 35 | 0.04 | 0.04 | 0.95 | 1.14 | 0.84 | 0.99 |
| 40 | 0.04 | 0.04 | 0.98 | 1.18 | 0.86 | 1.02 |
| 45 | 0.06 | 0.06 | 0.83 | 1 | 0.73 | 0.87 |
| 50 | 0.04 | 0.04 | 0.77 | 0.93 | 0.68 | 0.81 |
| 55 | 0.04 | 0.04 | 0.76 | 0.9 | 0.66 | 0.79 |
| 60 | 0.04 | 0.04 | 0.78 | 0.93 | 0.7 | 0.82 |
| 65 | 0.04 | 0.04 | 0.65 | 0.78 | 0.57 | 0.68 |
| 70 | 0.04 | 0.04 | 0.68 | 0.82 | 0.6 | 0.71 |
| MSE ($\times 10^3$) | | | | | | |
| 0 | 3.97 | 3.97 | 2.4 | 2.41 | 2.41 | 2.4 |
| 5 | 4.03 | 4.03 | 2.45 | 2.45 | 2.46 | 2.45 |
| 10 | 4.02 | 4.02 | 2.52 | 2.53 | 2.52 | 2.52 |
| 15 | 3.95 | 3.95 | 2.59 | 2.61 | 2.59 | 2.59 |
| 20 | 4.07 | 4.07 | 2.62 | 2.65 | 2.61 | 2.62 |
| 25 | 4.12 | 4.12 | 2.6 | 2.64 | 2.6 | 2.6 |
| 30 | 4.12 | 4.12 | 2.59 | 2.62 | 2.59 | 2.6 |
| 35 | 4.22 | 4.22 | 2.71 | 2.74 | 2.71 | 2.71 |
| 40 | 4.13 | 4.13 | 2.72 | 2.76 | 2.72 | 2.73 |
| 45 | 3.95 | 3.95 | 2.52 | 2.56 | 2.54 | 2.53 |
| 50 | 4.02 | 4.02 | 2.5 | 2.51 | 2.53 | 2.5 |
| 55 | 4.11 | 4.11 | 2.57 | 2.57 | 2.61 | 2.57 |
| 60 | 4 | 4 | 2.59 | 2.59 | 2.64 | 2.59 |
| 65 | 4.11 | 4.11 | 2.5 | 2.47 | 2.55 | 2.49 |
| 70 | 4.05 | 4.05 | 2.47 | 2.46 | 2.52 | 2.46 |
| Coverage | | | | | | |
| 0 | 0.94 | 0.94 | 0.95 | 0.95 | 0.95 | 0.95 |
| 5 | 0.94 | 0.94 | 0.95 | 0.95 | 0.95 | 0.95 |
| 10 | 0.95 | 0.95 | 0.95 | 0.95 | 0.95 | 0.95 |
| 15 | 0.94 | 0.94 | 0.95 | 0.95 | 0.95 | 0.95 |
| 20 | 0.94 | 0.94 | 0.95 | 0.95 | 0.95 | 0.95 |
| 25 | 0.94 | 0.94 | 0.95 | 0.95 | 0.95 | 0.95 |
| 30 | 0.94 | 0.94 | 0.95 | 0.95 | 0.95 | 0.95 |
| 35 | 0.94 | 0.94 | 0.95 | 0.95 | 0.95 | 0.95 |
| 40 | 0.94 | 0.94 | 0.95 | 0.95 | 0.95 | 0.95 |
| 45 | 0.94 | 0.94 | 0.95 | 0.95 | 0.95 | 0.95 |
| 50 | 0.94 | 0.94 | 0.95 | 0.95 | 0.95 | 0.95 |
| 55 | 0.94 | 0.94 | 0.95 | 0.95 | 0.95 | 0.95 |
| 60 | 0.94 | 0.94 | 0.95 | 0.95 | 0.95 | 0.95 |
| 65 | 0.94 | 0.94 | 0.94 | 0.95 | 0.95 | 0.95 |
| 70 | 0.94 | 0.94 | 0.95 | 0.94 | 0.95 | 0.95 |

**Table 2.** Simulation 1: Summary statistics of in-sample areas with a weak spatial structure ($A_1$)

| # Missing Districts | UNW | HT | NB | LN | AS | ES |
|---|---|---|---|---|---|---|
| Bias$^2$ ($\times 10^3$) | | | | | | |
| 5 | | | 1.24 | 1.24 | 1.51 | 1.24 |
| 10 | | | 4.49 | 4.47 | 5.65 | 4.49 |
| 15 | | | 7.69 | 7.68 | 8.21 | 7.69 |
| 20 | | | 8.55 | 8.53 | 9.35 | 8.55 |
| 25 | | | 10.85 | 10.84 | 11.68 | 10.85 |
| 30 | | | 10.94 | 10.93 | 11.82 | 10.94 |
| 35 | | | 10.2 | 10.19 | 10.93 | 10.2 |
| 40 | | | 10.79 | 10.8 | 11.46 | 10.79 |
| 45 | | | 10.23 | 10.24 | 10.46 | 10.23 |
| 50 | | | 10.19 | 10.21 | 10.19 | 10.19 |
| 55 | | | 9.4 | 9.42 | 9.36 | 9.4 |
| 60 | | | 9.03 | 9.05 | 9.01 | 9.03 |
| 65 | | | 9.25 | 9.27 | 9.25 | 9.26 |
| 70 | | | 9.26 | 9.28 | 9.11 | 9.26 |
| MSE ($\times 10^3$) | | | | | | |
| 5 | | | 1.21 | 1.19 | 1.12 | 1.21 |
| 10 | | | 4.37 | 4.37 | 5 | 4.37 |
| 15 | | | 7.64 | 7.65 | 8.05 | 7.64 |
| 20 | | | 8.57 | 8.58 | 9.39 | 8.57 |
| 25 | | | 10.42 | 10.43 | 11.01 | 10.43 |
| 30 | | | 10.39 | 10.38 | 10.92 | 10.39 |
| 35 | | | 9.86 | 9.87 | 10.2 | 9.86 |
| 40 | | | 10.24 | 10.26 | 10.52 | 10.25 |
| 45 | | | 10.31 | 10.32 | 10.61 | 10.31 |
| 50 | | | 10.25 | 10.28 | 10.33 | 10.26 |
| 55 | | | 9.46 | 9.48 | 9.51 | 9.47 |
| 60 | | | 9.09 | 9.11 | 9.16 | 9.1 |
| 65 | | | 9.33 | 9.35 | 9.42 | 9.34 |
| 70 | | | 9.33 | 9.34 | 9.28 | 9.34 |
| Coverage | | | | | | |
| 5 | | | 1 | 1 | 1 | 1 |
| 15 | | | 0.99 | 0.98 | 0.97 | 0.99 |
| 10 | | | 0.99 | 0.99 | 0.97 | 0.99 |
| 20 | | | 0.99 | 0.98 | 0.95 | 0.99 |
| 25 | | | 0.94 | 0.93 | 0.91 | 0.94 |
| 30 | | | 0.95 | 0.94 | 0.92 | 0.95 |
| 35 | | | 0.96 | 0.95 | 0.94 | 0.96 |
| 40 | | | 0.96 | 0.95 | 0.94 | 0.96 |
| 45 | | | 0.97 | 0.96 | 0.95 | 0.97 |
| 50 | | | 0.97 | 0.97 | 0.96 | 0.97 |
| 55 | | | 0.98 | 0.98 | 0.98 | 0.98 |
| 60 | | | 0.98 | 0.98 | 0.99 | 0.98 |
| 65 | | | 0.99 | 0.98 | 0.98 | 0.99 |
| 70 | | | 0.99 | 0.98 | 0.99 | 0.99 |

**Table 3.** Simulation 1: Summary statistics of off-sample areas with a weak spatial structure ($A_1$)

| # Missing Districts | UNW | HT | NB | LN | AS | ES |
|---|---|---|---|---|---|---|
| Bias$^2$ ($\times 10^3$) | | | | | | |
| 0 | 0.03 | 0.03 | 0.91 | 1.07 | 0.79 | 0.94 |
| 5 | 0.03 | 0.03 | 0.88 | 1.04 | 0.76 | 0.92 |
| 10 | 0.03 | 0.03 | 0.97 | 1.12 | 0.85 | 1 |
| 15 | 0.03 | 0.03 | 1.03 | 1.18 | 0.9 | 1.06 |
| 20 | 0.04 | 0.04 | 1.07 | 1.23 | 0.94 | 1.1 |
| 25 | 0.04 | 0.04 | 1.06 | 1.22 | 0.92 | 1.09 |
| 30 | 0.05 | 0.05 | 1.13 | 1.32 | 0.96 | 1.17 |
| 35 | 0.04 | 0.04 | 1.05 | 1.23 | 0.87 | 1.08 |
| 40 | 0.04 | 0.04 | 1.1 | 1.26 | 0.92 | 1.13 |
| 45 | 0.05 | 0.05 | 0.98 | 1.13 | 0.81 | 1.01 |
| 50 | 0.04 | 0.04 | 1.02 | 1.18 | 0.84 | 1.06 |
| 55 | 0.05 | 0.05 | 1.02 | 1.18 | 0.83 | 1.06 |
| 60 | 0.04 | 0.04 | 1.03 | 1.19 | 0.84 | 1.07 |
| 65 | 0.06 | 0.06 | 0.86 | 1.01 | 0.68 | 0.89 |
| 70 | 0.07 | 0.07 | 0.92 | 1.07 | 0.72 | 0.95 |
| MSE ($\times 10^3$) | | | | | | |
| 0 | 3.91 | 3.91 | 2.11 | 2.14 | 2.09 | 2.11 |
| 5 | 4.07 | 4.07 | 2.17 | 2.19 | 2.16 | 2.17 |
| 10 | 4.11 | 4.11 | 2.24 | 2.27 | 2.23 | 2.24 |
| 15 | 3.94 | 3.94 | 2.26 | 2.3 | 2.24 | 2.26 |
| 20 | 4.09 | 4.09 | 2.32 | 2.35 | 2.3 | 2.32 |
| 25 | 4.12 | 4.12 | 2.32 | 2.35 | 2.3 | 2.32 |
| 30 | 4.24 | 4.24 | 2.35 | 2.41 | 2.32 | 2.36 |
| 35 | 4.14 | 4.14 | 2.29 | 2.34 | 2.26 | 2.3 |
| 40 | 4.17 | 4.17 | 2.32 | 2.36 | 2.29 | 2.32 |
| 45 | 4.06 | 4.06 | 2.21 | 2.24 | 2.19 | 2.22 |
| 50 | 4.07 | 4.07 | 2.29 | 2.32 | 2.27 | 2.29 |
| 55 | 4.09 | 4.09 | 2.29 | 2.32 | 2.26 | 2.3 |
| 60 | 4.02 | 4.02 | 2.3 | 2.33 | 2.27 | 2.31 |
| 65 | 4.18 | 4.18 | 2.17 | 2.19 | 2.15 | 2.17 |
| 70 | 4.31 | 4.31 | 2.24 | 2.26 | 2.2 | 2.24 |
| Coverage | | | | | | |
| 0 | 0.94 | 0.94 | 0.95 | 0.94 | 0.95 | 0.95 |
| 5 | 0.94 | 0.94 | 0.94 | 0.94 | 0.95 | 0.94 |
| 10 | 0.94 | 0.94 | 0.94 | 0.94 | 0.95 | 0.94 |
| 15 | 0.94 | 0.94 | 0.94 | 0.94 | 0.95 | 0.94 |
| 20 | 0.94 | 0.94 | 0.94 | 0.93 | 0.94 | 0.94 |
| 25 | 0.95 | 0.95 | 0.94 | 0.94 | 0.95 | 0.94 |
| 30 | 0.94 | 0.94 | 0.94 | 0.93 | 0.94 | 0.94 |
| 35 | 0.94 | 0.94 | 0.94 | 0.94 | 0.94 | 0.94 |
| 40 | 0.94 | 0.94 | 0.94 | 0.94 | 0.94 | 0.94 |
| 45 | 0.94 | 0.94 | 0.94 | 0.94 | 0.95 | 0.95 |
| 50 | 0.94 | 0.94 | 0.94 | 0.94 | 0.94 | 0.94 |
| 55 | 0.94 | 0.94 | 0.94 | 0.94 | 0.95 | 0.94 |
| 60 | 0.94 | 0.94 | 0.94 | 0.94 | 0.95 | 0.94 |
| 65 | 0.94 | 0.94 | 0.94 | 0.94 | 0.95 | 0.95 |
| 70 | 0.94 | 0.94 | 0.94 | 0.94 | 0.95 | 0.94 |

**Table 4.** Simulation 1: Summary statistics of in-sample areas with a moderate spatial structure ($A_2$)

| # Missing Districts | UNW | HT | NB | LN | AS | ES |
|---|---|---|---|---|---|---|
| Bias$^2$ ($\times 10^3$) | | | | | | |
| 5 | | | 1.73 | 1.79 | 1.59 | 1.74 |
| 10 | | | 4.21 | 4.22 | 4.34 | 4.21 |
| 15 | | | 5.48 | 5.49 | 5.53 | 5.48 |
| 20 | | | 5.27 | 5.25 | 5.26 | 5.26 |
| 25 | | | 5.12 | 5.11 | 5.15 | 5.11 |
| 30 | | | 6.26 | 6.24 | 6.28 | 6.25 |
| 35 | | | 6.23 | 6.22 | 6.24 | 6.23 |
| 40 | | | 6.64 | 6.61 | 6.77 | 6.64 |
| 45 | | | 6.51 | 6.5 | 6.57 | 6.51 |
| 50 | | | 6.28 | 6.29 | 6.24 | 6.28 |
| 55 | | | 6.1 | 6.12 | 6.01 | 6.1 |
| 60 | | | 6.3 | 6.32 | 6.2 | 6.3 |
| 65 | | | 6.24 | 6.25 | 6.36 | 6.24 |
| 70 | | | 6.23 | 6.24 | 6.37 | 6.23 |
| MSE ($\times 10^3$) | | | | | | |
| 5 | | | 1.84 | 1.89 | 1.78 | 1.84 |
| 10 | | | 4.3 | 4.31 | 4.49 | 4.3 |
| 15 | | | 5.58 | 5.58 | 5.69 | 5.57 |
| 20 | | | 5.38 | 5.36 | 5.44 | 5.38 |
| 25 | | | 5.22 | 5.21 | 5.32 | 5.22 |
| 30 | | | 6.37 | 6.34 | 6.45 | 6.37 |
| 35 | | | 6.33 | 6.32 | 6.4 | 6.33 |
| 40 | | | 6.77 | 6.73 | 6.94 | 6.76 |
| 45 | | | 6.65 | 6.63 | 6.77 | 6.64 |
| 50 | | | 6.42 | 6.43 | 6.44 | 6.42 |
| 55 | | | 6.21 | 6.22 | 6.18 | 6.21 |
| 60 | | | 6.41 | 6.42 | 6.37 | 6.4 |
| 65 | | | 6.4 | 6.39 | 6.55 | 6.4 |
| 70 | | | 6.41 | 6.4 | 6.59 | 6.41 |
| Coverage | | | | | | |
| 5 | | | 1 | 1 | 1 | 1 |
| 10 | | | 1 | 1 | 1 | 1 |
| 15 | | | 0.97 | 0.96 | 0.97 | 0.97 |
| 20 | | | 0.97 | 0.97 | 0.97 | 0.97 |
| 25 | | | 0.98 | 0.97 | 0.98 | 0.98 |
| 30 | | | 0.94 | 0.93 | 0.94 | 0.94 |
| 35 | | | 0.95 | 0.94 | 0.95 | 0.95 |
| 40 | | | 0.95 | 0.95 | 0.95 | 0.95 |
| 45 | | | 0.92 | 0.92 | 0.94 | 0.92 |
| 50 | | | 0.94 | 0.93 | 0.95 | 0.94 |
| 55 | | | 0.95 | 0.94 | 0.97 | 0.95 |
| 60 | | | 0.95 | 0.95 | 0.96 | 0.95 |
| 65 | | | 0.96 | 0.95 | 0.97 | 0.95 |
| 70 | | | 0.96 | 0.95 | 0.98 | 0.96 |

**Table 5.** Simulation 1: Summary statistics of off-sample areas with a moderate spatial structure ($A_2$)

| # Missing Districts | UNW | HT | NB | LN | AS | ES |
|---|---|---|---|---|---|---|
| Bias$^2$ ($\times 10^3$) | | | | | | |
| 0 | 0.03 | 0.03 | 0.62 | 0.67 | 0.55 | 0.64 |
| 5 | 0.03 | 0.03 | 0.62 | 0.67 | 0.54 | 0.63 |
| 10 | 0.04 | 0.04 | 0.65 | 0.71 | 0.58 | 0.67 |
| 15 | 0.04 | 0.04 | 0.63 | 0.68 | 0.55 | 0.64 |
| 20 | 0.04 | 0.04 | 0.57 | 0.62 | 0.49 | 0.58 |
| 25 | 0.03 | 0.03 | 0.59 | 0.64 | 0.51 | 0.6 |
| 30 | 0.05 | 0.05 | 0.64 | 0.7 | 0.56 | 0.66 |
| 35 | 0.04 | 0.04 | 0.67 | 0.73 | 0.55 | 0.68 |
| 40 | 0.03 | 0.03 | 0.8 | 0.86 | 0.62 | 0.82 |
| 45 | 0.05 | 0.05 | 0.8 | 0.85 | 0.63 | 0.82 |
| 50 | 0.05 | 0.05 | 0.72 | 0.79 | 0.54 | 0.74 |
| 55 | 0.06 | 0.06 | 0.77 | 0.84 | 0.59 | 0.79 |
| 60 | 0.04 | 0.04 | 0.76 | 0.83 | 0.58 | 0.78 |
| 65 | 0.05 | 0.05 | 0.73 | 0.79 | 0.55 | 0.75 |
| 70 | 0.04 | 0.04 | 0.61 | 0.65 | 0.43 | 0.62 |
| MSE ($\times 10^3$) | | | | | | |
| 0 | 3.67 | 3.67 | 1.51 | 1.49 | 1.55 | 1.51 |
| 5 | 3.86 | 3.86 | 1.56 | 1.53 | 1.61 | 1.56 |
| 10 | 3.8 | 3.8 | 1.6 | 1.58 | 1.65 | 1.6 |
| 15 | 3.71 | 3.71 | 1.61 | 1.58 | 1.65 | 1.6 |
| 20 | 3.8 | 3.8 | 1.57 | 1.54 | 1.61 | 1.56 |
| 25 | 3.88 | 3.88 | 1.6 | 1.58 | 1.65 | 1.6 |
| 30 | 3.89 | 3.89 | 1.7 | 1.67 | 1.74 | 1.69 |
| 35 | 3.9 | 3.9 | 1.65 | 1.63 | 1.7 | 1.65 |
| 40 | 3.97 | 3.97 | 1.69 | 1.68 | 1.72 | 1.69 |
| 45 | 3.85 | 3.85 | 1.69 | 1.68 | 1.72 | 1.69 |
| 50 | 3.8 | 3.8 | 1.59 | 1.58 | 1.63 | 1.59 |
| 55 | 3.86 | 3.86 | 1.66 | 1.65 | 1.7 | 1.66 |
| 60 | 3.75 | 3.75 | 1.67 | 1.66 | 1.71 | 1.67 |
| 65 | 3.87 | 3.87 | 1.68 | 1.66 | 1.72 | 1.67 |
| 70 | 3.86 | 3.86 | 1.5 | 1.48 | 1.57 | 1.49 |
| Coverage | | | | | | |
| 0 | 0.95 | 0.95 | 0.95 | 0.95 | 0.95 | 0.95 |
| 5 | 0.94 | 0.94 | 0.95 | 0.95 | 0.95 | 0.95 |
| 10 | 0.94 | 0.94 | 0.95 | 0.95 | 0.95 | 0.95 |
| 15 | 0.94 | 0.94 | 0.95 | 0.95 | 0.95 | 0.95 |
| 20 | 0.95 | 0.95 | 0.95 | 0.95 | 0.95 | 0.95 |
| 25 | 0.95 | 0.95 | 0.95 | 0.95 | 0.96 | 0.95 |
| 30 | 0.95 | 0.95 | 0.95 | 0.95 | 0.95 | 0.95 |
| 35 | 0.94 | 0.94 | 0.95 | 0.94 | 0.95 | 0.95 |
| 40 | 0.94 | 0.94 | 0.94 | 0.94 | 0.95 | 0.94 |
| 45 | 0.94 | 0.94 | 0.95 | 0.95 | 0.96 | 0.95 |
| 50 | 0.94 | 0.94 | 0.95 | 0.95 | 0.96 | 0.95 |
| 55 | 0.94 | 0.94 | 0.95 | 0.95 | 0.96 | 0.95 |
| 60 | 0.95 | 0.95 | 0.95 | 0.95 | 0.96 | 0.95 |
| 65 | 0.94 | 0.94 | 0.95 | 0.95 | 0.96 | 0.95 |
| 70 | 0.94 | 0.94 | 0.96 | 0.96 | 0.96 | 0.96 |

**Table 6.** Simulation 1: Summary statistics of in-sample areas with a strong spatial structure ($A_3$)

| # Missing Districts | UNW | HT | NB | LN | AS | ES |
|---|---|---|---|---|---|---|
| Bias$^2$ ($\times 10^3$) | | | | | | |
| 5 | | | 1.57 | 1.62 | 1.58 | 1.57 |
| 10 | | | 2.03 | 2.08 | 2.09 | 2.03 |
| 15 | | | 1.97 | 2.02 | 2.06 | 1.97 |
| 20 | | | 2.64 | 2.67 | 2.73 | 2.64 |
| 25 | | | 2.54 | 2.59 | 2.63 | 2.54 |
| 30 | | | 2.36 | 2.41 | 2.44 | 2.36 |
| 35 | | | 3.97 | 3.96 | 3.94 | 3.97 |
| 40 | | | 4.8 | 4.8 | 4.84 | 4.81 |
| 45 | | | 4.54 | 4.54 | 4.57 | 4.54 |
| 50 | | | 4.69 | 4.7 | 4.7 | 4.69 |
| 55 | | | 4.47 | 4.46 | 4.48 | 4.47 |
| 60 | | | 4.28 | 4.27 | 4.29 | 4.28 |
| 65 | | | 4.22 | 4.23 | 4.22 | 4.22 |
| 70 | | | 5.09 | 5.1 | 5.03 | 5.09 |
| MSE ($\times 10^3$) | | | | | | |
| 5 | | | 1.94 | 1.97 | 1.89 | 1.94 |
| 10 | | | 2.34 | 2.37 | 2.35 | 2.33 |
| 15 | | | 2.31 | 2.33 | 2.34 | 2.31 |
| 20 | | | 2.96 | 2.98 | 3.01 | 2.96 |
| 25 | | | 2.83 | 2.86 | 2.88 | 2.83 |
| 30 | | | 2.69 | 2.71 | 2.72 | 2.68 |
| 35 | | | 4.28 | 4.25 | 4.2 | 4.28 |
| 40 | | | 5.1 | 5.08 | 5.1 | 5.1 |
| 45 | | | 4.84 | 4.83 | 4.82 | 4.84 |
| 50 | | | 4.97 | 4.96 | 4.94 | 4.97 |
| 55 | | | 4.74 | 4.72 | 4.72 | 4.74 |
| 60 | | | 4.6 | 4.57 | 4.56 | 4.6 |
| 65 | | | 4.56 | 4.55 | 4.51 | 4.56 |
| 70 | | | 5.46 | 5.44 | 5.34 | 5.45 |
| Coverage | | | | | | |
| 5 | | | 1 | 1 | 1 | 1 |
| 10 | | | 1 | 1 | 1 | 1 |
| 15 | | | 1 | 1 | 1 | 1 |
| 20 | | | 1 | 1 | 1 | 1 |
| 25 | | | 0.98 | 0.98 | 0.99 | 0.98 |
| 30 | | | 0.99 | 0.98 | 0.99 | 0.99 |
| 35 | | | 0.95 | 0.94 | 0.95 | 0.95 |
| 40 | | | 0.91 | 0.9 | 0.92 | 0.9 |
| 45 | | | 0.92 | 0.91 | 0.94 | 0.92 |
| 50 | | | 0.91 | 0.91 | 0.95 | 0.91 |
| 55 | | | 0.92 | 0.92 | 0.95 | 0.92 |
| 60 | | | 0.93 | 0.93 | 0.96 | 0.93 |
| 65 | | | 0.94 | 0.94 | 0.97 | 0.94 |
| 70 | | | 0.94 | 0.93 | 0.97 | 0.94 |

**Table 7.** Simulation 1: Summary statistics of off-sample areas with a strong spatial structure ($A_3$)

| Scenario (B) | Scenario (A) | UNW | HT | NB | LN | AN | ES |
|---|---|---|---|---|---|---|---|
| Bias$^2$ ($\times 10^3$) | | | | | | | |
| 1 | 1 | 0.04 | 0.04 | 1.07 | 1.88 | 1.71 | 1.75 |
| 1 | 2 | 0.03 | 0.04 | 0.60 | 0.93 | 0.80 | 0.90 |
| 1 | 3 | 0.02 | 0.02 | 0.54 | 0.57 | 0.39 | 0.53 |
| 2 | 1 | 0.14 | 0.34 | 0.81 | 1.93 | 1.84 | 1.83 |
| 2 | 2 | 0.14 | 0.37 | 0.34 | 0.85 | 0.86 | 0.85 |
| 2 | 3 | 0.09 | 0.36 | 0.39 | 0.82 | 0.68 | 0.83 |
| Variance ($\times 10^3$) | | | | | | | |
| 1 | 1 | 2.98 | 3.56 | 1.46 | 1.90 | 2.11 | 1.96 |
| 1 | 2 | 2.97 | 3.53 | 1.09 | 1.48 | 1.60 | 1.51 |
| 1 | 3 | 2.67 | 3.13 | 0.91 | 1.48 | 1.65 | 1.51 |
| 2 | 1 | 3 | 3.58 | 1.49 | 1.77 | 1.82 | 1.78 |
| 2 | 2 | 2.88 | 3.50 | 1.42 | 1.79 | 1.79 | 1.80 |
| 2 | 3 | 2.60 | 3.15 | 1.30 | 1.77 | 1.88 | 1.79 |
| MSE ($\times 10^3$) | | | | | | | |
| 1 | 1 | 3.01 | 3.60 | 2.54 | 3.78 | 3.81 | 3.71 |
| 1 | 2 | 3 | 3.57 | 1.69 | 2.41 | 2.40 | 2.41 |
| 1 | 3 | 2.69 | 3.16 | 1.45 | 2.05 | 2.04 | 2.03 |
| 2 | 1 | 3.14 | 3.91 | 2.30 | 3.69 | 3.66 | 3.61 |
| 2 | 2 | 3.02 | 3.87 | 1.76 | 2.65 | 2.65 | 2.65 |
| 2 | 3 | 2.69 | 3.51 | 1.69 | 2.59 | 2.56 | 2.62 |
| Coverage | | | | | | | |
| 1 | 1 | 0.94 | 0.93 | 0.94 | 0.81 | 0.81 | 0.81 |
| 1 | 2 | 0.94 | 0.94 | 0.95 | 0.83 | 0.83 | 0.82 |
| 1 | 3 | 0.94 | 0.94 | 0.95 | 0.83 | 0.83 | 0.83 |
| 2 | 1 | 0.95 | 0.93 | 0.95 | 0.80 | 0.80 | 0.80 |
| 2 | 2 | 0.95 | 0.94 | 0.96 | 0.81 | 0.81 | 0.81 |
| 2 | 3 | 0.95 | 0.92 | 0.96 | 0.81 | 0.80 | 0.80 |
| Average Length CI | | | | | | | |
| 1 | 1 | 0.20 | 0.22 | 0.17 | 0.17 | 0.17 | 0.17 |
| 1 | 2 | 0.20 | 0.22 | 0.16 | 0.16 | 0.16 | 0.16 |
| 1 | 3 | 0.19 | 0.21 | 0.14 | 0.14 | 0.15 | 0.14 |
| 2 | 1 | 0.20 | 0.22 | 0.17 | 0.17 | 0.17 | 0.17 |
| 2 | 2 | 0.20 | 0.22 | 0.17 | 0.18 | 0.17 | 0.17 |
| 2 | 3 | 0.19 | 0.21 | 0.16 | 0.17 | 0.17 | 0.17 |

**Table 8.** Simulation 2: Summary Statistics for the in-sample areas for model ($M0$), not taking into account the auxiliary data

| Scenario (B) | Scenario (A) | UNW | HT | NB | LN | AN | ES |
|---|---|---|---|---|---|---|---|
| | | | Bias$^2$ ($\times 10^3$) | | | | |
| 1 | 1 | | | 7.06 | 6.86 | 6.87 | 6.95 |
| 1 | 2 | | | 4.91 | 5.01 | 5.16 | 4.95 |
| 1 | 3 | | | 2.86 | 3.06 | 2.86 | 3.04 |
| 2 | 1 | | | 10.11 | 9.28 | 9.86 | 9.98 |
| 2 | 2 | | | 9.08 | 7.96 | 8.38 | 8.58 |
| 2 | 3 | | | 7.12 | 7.26 | 7.20 | 7.99 |
| | | | Variance ($\times 10^3$) | | | | |
| 1 | 1 | | | 0.05 | 0.08 | 0.17 | 0.08 |
| 1 | 2 | | | 0.09 | 0.11 | 0.15 | 0.11 |
| 1 | 3 | | | 0.25 | 0.34 | 0.28 | 0.33 |
| 2 | 1 | | | 0.10 | 0.13 | 0.17 | 0.12 |
| 2 | 2 | | | 0.20 | 0.23 | 0.22 | 0.23 |
| 2 | 3 | | | 0.23 | 0.28 | 0.26 | 0.27 |
| | | | MSE ($\times 10^3$) | | | | |
| 1 | 1 | | | 7.11 | 6.94 | 7.04 | 7.04 |
| 1 | 2 | | | 5.00 | 5.12 | 5.31 | 5.06 |
| 1 | 3 | | | 3.11 | 3.40 | 3.15 | 3.37 |
| 2 | 1 | | | 10.22 | 9.41 | 10.03 | 10.10 |
| 2 | 2 | | | 9.28 | 8.20 | 8.60 | 8.81 |
| 2 | 3 | | | 7.35 | 7.54 | 7.46 | 8.26 |
| | | | Coverage | | | | |
| 1 | 1 | | | 0.96 | 0.97 | 0.98 | 0.98 |
| 1 | 2 | | | 0.96 | 0.99 | 0.99 | 0.99 |
| 1 | 3 | | | 0.99 | 0.99 | 1 | 0.99 |
| 2 | 1 | | | 0.94 | 0.94 | 0.95 | 0.94 |
| 2 | 2 | | | 0.97 | 0.99 | 0.99 | 0.99 |
| 2 | 3 | | | 0.97 | 0.98 | 1 | 0.98 |
| | | | Average Length CI | | | | |
| 1 | 1 | | | 0.38 | 0.39 | 0.41 | 0.39 |
| 1 | 2 | | | 0.29 | 0.32 | 0.34 | 0.32 |
| 1 | 3 | | | 0.26 | 0.30 | 0.33 | 0.30 |
| 2 | 1 | | | 0.38 | 0.39 | 0.40 | 0.39 |
| 2 | 2 | | | 0.38 | 0.41 | 0.40 | 0.41 |
| 2 | 3 | | | 0.38 | 0.41 | 0.43 | 0.41 |

**Table 9.** Simulation 2: Summary Statistics for the off-sample areas for model ($M0$), not taking into account the auxiliary data

| Scenario (B) | Scenario (A) | UNW | HT | NB | LN | AN | ES |
|---|---|---|---|---|---|---|---|
| | | Bias$^2$ ($\times 10^3$) | | | | | |
| 1 | 1 | 5.83 | 5.84 | 9.30 | 9.74 | 10.65 | 10.50 |
| 1 | 2 | 7.74 | 7.73 | 5.77 | 5.72 | 6.01 | 5.88 |
| 1 | 3 | 9.15 | 9.23 | 18.53 | 20.44 | 19.64 | 20.08 |
| 2 | 1 | 5.62 | 5.60 | 9.04 | 9.22 | 9.98 | 9.85 |
| 2 | 2 | 7.33 | 7.36 | 6.14 | 6.11 | 6.31 | 6.24 |
| 2 | 3 | 8.65 | 8.65 | 18.99 | 20.31 | 20.03 | 20.28 |
| | | Variance ($\times 10^3$) | | | | | |
| 1 | 1 | 3.46 | 3.95 | 0.05 | 0.18 | 0.27 | 0.25 |
| 1 | 2 | 3.45 | 3.96 | 0.05 | 0.29 | 0.32 | 0.32 |
| 1 | 3 | 3.22 | 3.64 | 0.05 | 0.17 | 0.45 | 0.37 |
| 2 | 1 | 3.51 | 4.04 | 0.05 | 0.15 | 0.21 | 0.20 |
| 2 | 2 | 3.43 | 3.94 | 0.05 | 0.22 | 0.22 | 0.23 |
| 2 | 3 | 3.20 | 3.70 | 0.06 | 0.18 | 0.38 | 0.32 |
| | | MSE ($\times 10^3$) | | | | | |
| 1 | 1 | 9.29 | 9.79 | 9.35 | 9.91 | 10.91 | 10.75 |
| 1 | 2 | 11.19 | 11.69 | 5.82 | 6.01 | 6.33 | 6.20 |
| 1 | 3 | 12.37 | 12.88 | 18.58 | 20.61 | 20.09 | 20.45 |
| 2 | 1 | 9.13 | 9.64 | 9.09 | 9.37 | 10.19 | 10.04 |
| 2 | 2 | 10.76 | 11.31 | 6.19 | 6.33 | 6.54 | 6.47 |
| 2 | 3 | 11.86 | 12.34 | 19.04 | 20.49 | 20.41 | 20.59 |
| | | Coverage | | | | | |
| 1 | 1 | 0.82 | 0.83 | 0.14 | 0.12 | 0.14 | 0.13 |
| 1 | 2 | 0.81 | 0.81 | 0.19 | 0.14 | 0.16 | 0.14 |
| 1 | 3 | 0.83 | 0.83 | 0.03 | 0.05 | 0.06 | 0.05 |
| 2 | 1 | 0.81 | 0.82 | 0.14 | 0.13 | 0.14 | 0.13 |
| 2 | 2 | 0.80 | 0.80 | 0.19 | 0.15 | 0.16 | 0.15 |
| 2 | 3 | 0.82 | 0.83 | 0.04 | 0.04 | 0.06 | 0.06 |
| | | Average Length CI | | | | | |
| 1 | 1 | 0.21 | 0.23 | 0.03 | 0.02 | 0.03 | 0.02 |
| 1 | 2 | 0.21 | 0.22 | 0.03 | 0.02 | 0.03 | 0.02 |
| 1 | 3 | 0.20 | 0.22 | 0.03 | 0.03 | 0.03 | 0.02 |
| 2 | 1 | 0.22 | 0.23 | 0.03 | 0.03 | 0.03 | 0.02 |
| 2 | 2 | 0.22 | 0.23 | 0.03 | 0.03 | 0.03 | 0.02 |
| 2 | 3 | 0.21 | 0.22 | 0.03 | 0.03 | 0.03 | 0.02 |

**Table 10.** Simulation 2: Summary Statistics for the in-sample areas for model $(M1)$. The simulated data was analysed using “energy” as a covariate.

| Scenario (B) | Scenario (A) | UNW | HT | NB | LN | AN | ES |
|---|---|---|---|---|---|---|---|
| | | | Bias$^2$ ($\times 10^3$) | | | | |
| 1 | 1 | | | 7.05 | 6.92 | 7.18 | 7.03 |
| 1 | 2 | | | 5.33 | 5.37 | 5.62 | 5.47 |
| 1 | 3 | | | 17.56 | 19.14 | 17.82 | 18.10 |
| 2 | 1 | | | 7.69 | 7.59 | 7.76 | 7.67 |
| 2 | 2 | | | 5.95 | 5.98 | 6.13 | 6.05 |
| 2 | 3 | | | 19.20 | 20.47 | 19.85 | 19.94 |
| | | | Variance ($\times 10^3$) | | | | |
| 1 | 1 | | | 0.06 | 0.18 | 0.25 | 0.24 |
| 1 | 2 | | | 0.06 | 0.28 | 0.31 | 0.31 |
| 1 | 3 | | | 0.06 | 0.18 | 0.42 | 0.34 |
| 2 | 1 | | | 0.06 | 0.16 | 0.21 | 0.20 |
| 2 | 2 | | | 0.06 | 0.21 | 0.23 | 0.23 |
| 2 | 3 | | | 0.07 | 0.18 | 0.35 | 0.29 |
| | | | MSE ($\times 10^3$) | | | | |
| 1 | 1 | | | 7.11 | 7.10 | 7.44 | 7.26 |
| 1 | 2 | | | 5.39 | 5.66 | 5.94 | 5.78 |
| 1 | 3 | | | 17.62 | 19.32 | 18.24 | 18.44 |
| 2 | 1 | | | 7.75 | 7.75 | 7.97 | 7.87 |
| 2 | 2 | | | 6.01 | 6.20 | 6.36 | 6.28 |
| 2 | 3 | | | 19.27 | 20.64 | 20.20 | 20.23 |
| | | | Coverage | | | | |
| 1 | 1 | | | 0.13 | 0.14 | 0.15 | 0.14 |
| 1 | 2 | | | 0.12 | 0.13 | 0.12 | 0.11 |
| 1 | 3 | | | 0.09 | 0.07 | 0.06 | 0.06 |
| 2 | 1 | | | 0.13 | 0.13 | 0.14 | 0.13 |
| 2 | 2 | | | 0.11 | 0.11 | 0.12 | 0.11 |
| 2 | 3 | | | 0.09 | 0.07 | 0.06 | 0.05 |
| | | | Av Length CI | | | | |
| 1 | 1 | | | 0.03 | 0.03 | 0.03 | 0.03 |
| 1 | 2 | | | 0.03 | 0.03 | 0.03 | 0.03 |
| 1 | 3 | | | 0.03 | 0.03 | 0.03 | 0.03 |
| 2 | 1 | | | 0.03 | 0.03 | 0.03 | 0.03 |
| 2 | 2 | | | 0.03 | 0.03 | 0.03 | 0.03 |
| 2 | 3 | | | 0.03 | 0.03 | 0.03 | 0.03 |

**Table 11.** Simulation 2: Summary Statistics for the off-sample areas for model ($M1$). The simulated data was analysed using "energy" as a covariate.

| Scenario (B) | Scenario (A) | UNW | HT | NB | LN | AN | ES |
|---|---|---|---|---|---|---|---|
| | | Bias$^2$ ($\times 10^3$) | | | | | |
| 1 | 1 | 5.83 | 5.84 | 1.07 | 2.56 | 2.36 | 2.42 |
| 1 | 2 | 7.74 | 7.73 | 0.60 | 1.14 | 0.92 | 1.04 |
| 1 | 3 | 9.15 | 9.23 | 0.54 | 1.30 | 0.70 | 0.90 |
| 2 | 1 | 5.62 | 5.60 | 3.09 | 6.20 | 7.29 | 6.84 |
| 2 | 2 | 7.33 | 7.36 | 2.03 | 4.79 | 6.23 | 5.66 |
| 2 | 3 | 8.65 | 8.65 | 2.72 | 5.13 | 5.99 | 5.66 |
| | | Variance ($\times 10^3$) | | | | | |
| 1 | 1 | 3.46 | 3.95 | 1.46 | 1.90 | 2.19 | 2.06 |
| 1 | 2 | 3.45 | 3.96 | 1.09 | 1.66 | 1.89 | 1.78 |
| 1 | 3 | 3.22 | 3.64 | 0.91 | 1.51 | 1.80 | 1.65 |
| 2 | 1 | 3.51 | 4.04 | 1.49 | 1.94 | 2.30 | 2.27 |
| 2 | 2 | 3.43 | 3.94 | 1.42 | 1.81 | 2.12 | 2.09 |
| 2 | 3 | 3.20 | 3.70 | 1.31 | 1.81 | 2.14 | 2.02 |
| | | MSE ($\times 10^3$) | | | | | |
| 1 | 1 | 9.29 | 9.79 | 2.53 | 4.46 | 4.55 | 4.47 |
| 1 | 2 | 11.19 | 11.69 | 1.69 | 2.80 | 2.81 | 2.81 |
| 1 | 3 | 12.37 | 12.88 | 1.46 | 2.81 | 2.50 | 2.55 |
| 2 | 1 | 9.13 | 9.64 | 4.59 | 8.15 | 9.59 | 9.12 |
| 2 | 2 | 10.76 | 11.31 | 3.45 | 6.60 | 8.35 | 7.75 |
| 2 | 3 | 11.86 | 12.34 | 4.03 | 6.95 | 8.13 | 7.68 |
| | | Coverage | | | | | |
| 1 | 1 | 0.82 | 0.83 | 0.84 | 0.71 | 0.70 | 0.70 |
| 1 | 2 | 0.81 | 0.81 | 0.84 | 0.73 | 0.73 | 0.73 |
| 1 | 3 | 0.83 | 0.83 | 0.83 | 0.69 | 0.73 | 0.71 |
| 2 | 1 | 0.81 | 0.82 | 0.69 | 0.63 | 0.61 | 0.62 |
| 2 | 2 | 0.80 | 0.80 | 0.68 | 0.67 | 0.62 | 0.63 |
| 2 | 3 | 0.82 | 0.83 | 0.69 | 0.65 | 0.63 | 0.64 |
| | | Average Length CI | | | | | |
| 1 | 1 | 0.21 | 0.23 | 0.12 | 0.14 | 0.14 | 0.14 |
| 1 | 2 | 0.21 | 0.22 | 0.11 | 0.13 | 0.13 | 0.13 |
| 1 | 3 | 0.20 | 0.22 | 0.10 | 0.12 | 0.12 | 0.12 |
| 2 | 1 | 0.22 | 0.23 | 0.12 | 0.15 | 0.15 | 0.15 |
| 2 | 2 | 0.22 | 0.23 | 0.12 | 0.15 | 0.15 | 0.15 |
| 2 | 3 | 0.21 | 0.22 | 0.12 | 0.14 | 0.14 | 0.14 |

**Table 12.** Simulation 2: Summary Statistics for the in-sample areas for model ($M2$). The simulated data was analysed using the random effects $u_k^*$ & $v_k^*$.

| Scenario (B) | Scenario (A) | UNW | HT | UWB | LN | AN | ESS |
|---|---|---|---|---|---|---|---|
| Bias$^2$ ($\times 10^3$) | | | | | | | |
| 1 | 1 | | | 7.07 | 7.16 | 6.94 | 6.98 |
| 1 | 2 | | | 4.91 | 4.98 | 5.13 | 4.94 |
| 1 | 3 | | | 2.86 | 3.95 | 3.24 | 3.41 |
| 2 | 1 | | | 9.99 | 10.02 | 11.25 | 10.80 |
| 2 | 2 | | | 8.30 | 9.00 | 10.99 | 10.24 |
| 2 | 3 | | | 5.70 | 7.28 | 8.01 | 7.73 |
| Variance ($\times 10^3$) | | | | | | | |
| 1 | 1 | | | 0.06 | 0.09 | 0.18 | 0.09 |
| 1 | 2 | | | 0.09 | 0.14 | 0.19 | 0.14 |
| 1 | 3 | | | 0.25 | 0.34 | 0.31 | 0.35 |
| 2 | 1 | | | 0.10 | 0.12 | 0.24 | 0.14 |
| 2 | 2 | | | 0.21 | 0.27 | 0.31 | 0.35 |
| 2 | 3 | | | 0.23 | 0.27 | 0.27 | 0.29 |
| MSE ($\times 10^3$) | | | | | | | |
| 1 | 1 | | | 7.13 | 7.25 | 7.11 | 7.07 |
| 1 | 2 | | | 5.00 | 5.12 | 5.32 | 5.09 |
| 1 | 3 | | | 3.11 | 4.29 | 3.54 | 3.76 |
| 2 | 1 | | | 10.10 | 10.14 | 11.49 | 10.94 |
| 2 | 2 | | | 8.50 | 9.28 | 11.30 | 10.59 |
| 2 | 3 | | | 5.93 | 7.55 | 8.28 | 8.02 |
| Coverage | | | | | | | |
| 1 | 1 | | | 0.87 | 0.89 | 0.91 | 0.90 |
| 1 | 2 | | | 0.83 | 0.87 | 0.90 | 0.88 |
| 1 | 3 | | | 0.89 | 0.87 | 0.98 | 0.92 |
| 2 | 1 | | | 0.86 | 0.89 | 0.89 | 0.90 |
| 2 | 2 | | | 0.89 | 0.91 | 0.90 | 0.91 |
| 2 | 3 | | | 0.94 | 0.97 | 0.96 | 0.97 |
| Average Length CI | | | | | | | |
| 1 | 1 | | | 0.28 | 0.29 | 0.31 | 0.29 |
| 1 | 2 | | | 0.22 | 0.24 | 0.26 | 0.25 |
| 1 | 3 | | | 0.19 | 0.22 | 0.25 | 0.23 |
| 2 | 1 | | | 0.28 | 0.33 | 0.34 | 0.35 |
| 2 | 2 | | | 0.28 | 0.31 | 0.33 | 0.34 |
| 2 | 3 | | | 0.27 | 0.32 | 0.34 | 0.33 |

**Table 13.** Simulation 2: Summary Statistics for the off-sample areas for model ($M2$). The simulated data was analysed using the random effects $u_k^*$ & $v_k^*$.

| Scenario (B) | Scenario (A) | UNW | HT | NB | LN | AN | ES |
|---|---|---|---|---|---|---|---|
| | | Bias$^2$ ($\times 10^3$) | | | | | |
| 1 | 1 | 5.83 | 5.84 | 1.07 | 2.54 | 2.34 | 2.38 |
| 1 | 2 | 7.74 | 7.73 | 0.60 | 1.12 | 0.91 | 1.01 |
| 1 | 3 | 9.15 | 9.23 | 0.54 | 1.26 | 0.66 | 0.84 |
| 2 | 1 | 5.62 | 5.60 | 1.12 | 2.46 | 2.58 | 2.25 |
| 2 | 2 | 7.33 | 7.36 | 0.65 | 1.21 | 1.20 | 1.06 |
| 2 | 3 | 8.65 | 8.65 | 0.63 | 1.38 | 1.03 | 1.00 |
| | | Variance ($\times 10^3$) | | | | | |
| 1 | 1 | 3.46 | 3.95 | 1.48 | 2.08 | 2.35 | 2.25 |
| 1 | 2 | 3.45 | 3.96 | 1.11 | 1.85 | 2.06 | 1.99 |
| 1 | 3 | 3.22 | 3.64 | 0.94 | 1.72 | 1.98 | 1.83 |
| 2 | 1 | 3.51 | 4.04 | 1.52 | 2.30 | 2.24 | 2.53 |
| 2 | 2 | 3.43 | 3.94 | 1.12 | 1.98 | 1.95 | 2.20 |
| 2 | 3 | 3.20 | 3.70 | 0.93 | 1.84 | 1.93 | 1.99 |
| | | MSE ($\times 10^3$) | | | | | |
| 1 | 1 | 9.29 | 9.79 | 2.55 | 4.62 | 4.69 | 4.62 |
| 1 | 2 | 11.19 | 11.69 | 1.72 | 2.96 | 2.97 | 3.00 |
| 1 | 3 | 12.37 | 12.88 | 1.48 | 2.98 | 2.64 | 2.66 |
| 2 | 1 | 9.13 | 9.64 | 2.64 | 4.76 | 4.82 | 4.78 |
| 2 | 2 | 10.76 | 11.31 | 1.76 | 3.19 | 3.15 | 3.26 |
| 2 | 3 | 11.86 | 12.34 | 1.56 | 3.22 | 2.95 | 2.99 |
| | | Coverage | | | | | |
| 1 | 1 | 0.82 | 0.83 | 0.85 | 0.72 | 0.71 | 0.71 |
| 1 | 2 | 0.81 | 0.81 | 0.85 | 0.74 | 0.74 | 0.74 |
| 1 | 3 | 0.83 | 0.83 | 0.84 | 0.70 | 0.74 | 0.72 |
| 2 | 1 | 0.81 | 0.82 | 0.85 | 0.72 | 0.70 | 0.72 |
| 2 | 2 | 0.80 | 0.80 | 0.86 | 0.75 | 0.73 | 0.75 |
| 2 | 3 | 0.82 | 0.83 | 0.86 | 0.72 | 0.73 | 0.74 |
| | | Average Length CI | | | | | |
| 1 | 1 | 0.21 | 0.23 | 0.13 | 0.14 | 0.14 | 0.14 |
| 1 | 2 | 0.21 | 0.22 | 0.12 | 0.13 | 0.13 | 0.13 |
| 1 | 3 | 0.20 | 0.22 | 0.11 | 0.12 | 0.13 | 0.12 |
| 2 | 1 | 0.22 | 0.23 | 0.13 | 0.15 | 0.14 | 0.15 |
| 2 | 2 | 0.22 | 0.23 | 0.12 | 0.14 | 0.13 | 0.14 |
| 2 | 3 | 0.21 | 0.22 | 0.11 | 0.13 | 0.13 | 0.13 |

**Table 14.** Simulation 2: Summary Statistics for the in-sample areas for model ($M3$). The simulated data was analysed using the random effects $u_k^*$ & $v_k^*$ and "energy" as a covariate.

| Scenario (B) | Scenario (A) | UNW | HT | NB | LN | AN | ES |
|---|---|---|---|---|---|---|---|
| | | | Bias$^2$ ($\times 10^3$) | | | | |
| 1 | 1 | | | 7.08 | 7.19 | 6.88 | 6.94 |
| 1 | 2 | | | 4.89 | 4.99 | 5.12 | 4.94 |
| 1 | 3 | | | 2.88 | 4.00 | 3.25 | 3.38 |
| 2 | 1 | | | 7.75 | 7.80 | 7.49 | 7.64 |
| 2 | 2 | | | 5.44 | 5.54 | 5.66 | 5.49 |
| 2 | 3 | | | 3.17 | 4.34 | 3.90 | 3.75 |
| | | | Variance ($\times 10^3$) | | | | |
| 1 | 1 | | | 0.09 | 0.18 | 0.27 | 0.20 |
| 1 | 2 | | | 0.12 | 0.27 | 0.32 | 0.27 |
| 1 | 3 | | | 0.28 | 0.48 | 0.44 | 0.51 |
| 2 | 1 | | | 0.09 | 0.19 | 0.31 | 0.21 |
| 2 | 2 | | | 0.15 | 0.27 | 0.35 | 0.28 |
| 2 | 3 | | | 0.27 | 0.44 | 0.41 | 0.50 |
| | | | MSE ($\times 10^3$) | | | | |
| 1 | 1 | | | 7.16 | 7.37 | 7.15 | 7.14 |
| 1 | 2 | | | 5.01 | 5.26 | 5.44 | 5.21 |
| 1 | 3 | | | 3.16 | 4.48 | 3.69 | 3.89 |
| 2 | 1 | | | 7.84 | 7.99 | 7.81 | 7.85 |
| 2 | 2 | | | 5.59 | 5.82 | 6.01 | 5.78 |
| 2 | 3 | | | 3.44 | 4.79 | 4.30 | 4.25 |
| | | | Coverage | | | | |
| 1 | 1 | | | 0.87 | 0.89 | 0.92 | 0.91 |
| 1 | 2 | | | 0.83 | 0.88 | 0.90 | 0.88 |
| 1 | 3 | | | 0.89 | 0.88 | 0.98 | 0.92 |
| 2 | 1 | | | 0.86 | 0.90 | 0.88 | 0.91 |
| 2 | 2 | | | 0.83 | 0.87 | 0.86 | 0.89 |
| 2 | 3 | | | 0.90 | 0.91 | 0.97 | 0.94 |
| | | | Average Length CI | | | | |
| 1 | 1 | | | 0.28 | 0.30 | 0.31 | 0.30 |
| 1 | 2 | | | 0.22 | 0.25 | 0.27 | 0.25 |
| 1 | 3 | | | 0.19 | 0.23 | 0.25 | 0.23 |
| 2 | 1 | | | 0.29 | 0.31 | 0.29 | 0.32 |
| 2 | 2 | | | 0.23 | 0.26 | 0.26 | 0.27 |
| 2 | 3 | | | 0.20 | 0.24 | 0.26 | 0.25 |

**Table 15.** Simulation 2: Summary Statistics for the off-sample areas for model ($M3$). The simulated data was analysed using the random effects $u_k^*$ & $v_k^*$ and "energy" as a covariate.

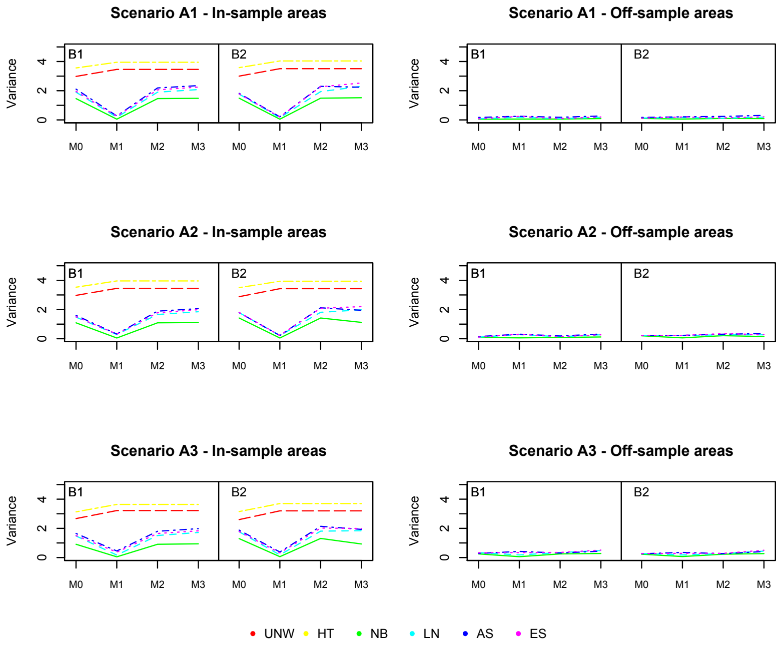


**Figure 2.** Simulation 2: Variance

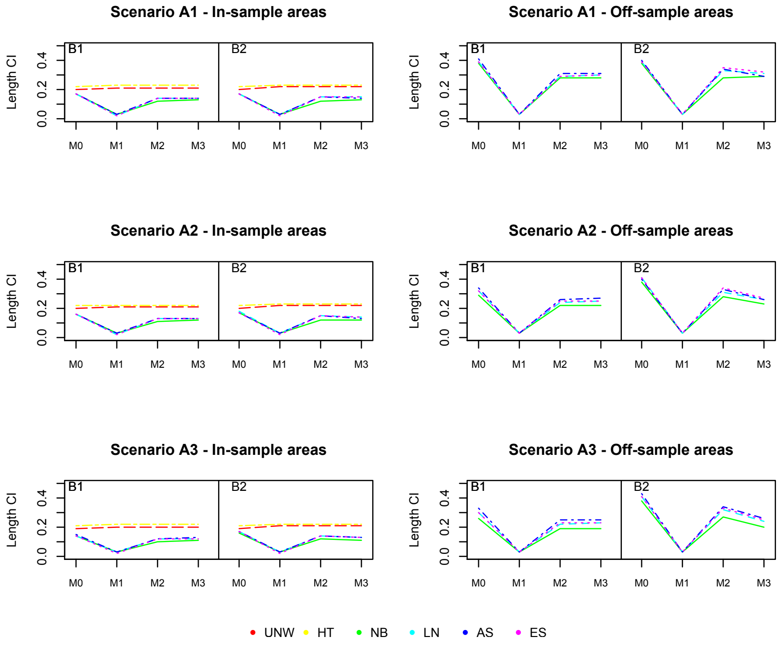


**Figure 3.** Simulation 2: Length Confidence Intervals
